# Design of Metaheuristic Optimization-Based Vascular Segmentation Techniques for Photoacoustic Images

**DOI:** 10.1155/2022/4736113

**Published:** 2022-01-30

**Authors:** Thavavel Vaiyapuri, Ashit Kumar Dutta, Mohamed Yacin Sikkandar, Deepak Gupta, Bader Alouffi, Abdullah Alharbi, Hafiz Tayyab Rauf, Seifedine Kadry

**Affiliations:** ^1^College of Computer Engineering and Sciences, Prince Sattam Bin Abdulaziz University, Al-Kharj, Saudi Arabia; ^2^Department of Computer Science and Information Systems, College of Applied Sciences, AlMaarefa University, Ad Diriyah, Riyadh 13713, Saudi Arabia; ^3^Department of Medical Equipment Technology, College of Applied Medical Sciences, Majmaah University, Al Majmaah 11952, Saudi Arabia; ^4^Department of Computer Science & Engineering, Maharaja Agrasen Institute of Technology, Delhi, India; ^5^Department of Computer Science, College of Computers and Information Technology, Taif University, P.O. Box 11099, Taif 21944, Saudi Arabia; ^6^Department of Information Technology, College of Computers and Information Technology, Taif University, P.O. Box 11099, Taif 21944, Saudi Arabia; ^7^Department of Computer Science, Faculty of Engineering & Informatics, University of Bradford, Bradford, UK; ^8^Faculty of Applied Computing and Technology, Noroff University College, Kristiansand, Norway

## Abstract

Biomedical imaging technologies are designed to offer functional, anatomical, and molecular details related to the internal organs. Photoacoustic imaging (PAI) is becoming familiar among researchers and industrialists. The PAI is found useful in several applications of brain and cancer imaging such as prostate cancer, breast cancer, and ovarian cancer. At the same time, the vessel images hold important medical details which offer strategies for a qualified diagnosis. Recently developed image processing techniques can be employed to segment vessels. Since vessel segmentation on PAI is a difficult process, this paper employs metaheuristic optimization-based vascular segmentation techniques for PAI. The proposed model involves two distinct kinds of vessel segmentation approaches such as Shannon's entropy function (SEF) and multilevel Otsu thresholding (MLOT). Moreover, the threshold value and entropy function in the segmentation process are optimized using three metaheuristics such as the cuckoo search (CS), equilibrium optimizer (EO), and harmony search (HS) algorithms. A detailed experimental analysis is made on benchmark PAI dataset, and the results are inspected under varying aspects. The obtained results pointed out the supremacy of the presented model with a higher accuracy of 98.71%.

## 1. Introduction

Photoacoustic tomography (PAT) is a kind of hybrid imaging approach which integrates the advantages of both ultrasonic and optical imaging. In PAT, ultrasonic waves are stimulated by the pulsed laser that has embodied both ultrasonic deep penetration and optical absorption contrast [1]. Various real-time applications are being studied to demonstrate their great opportunities in both clinical and preclinical imaging, such as breast cancer diagnostics and small animal entire body imaging [2]. In addition, multispectral PAT has exclusive benefits from monitoring the functional data of biological tissues, for example, metabolism and blood oxygen saturation (sO2). Especially, photoacoustic computed tomography (PACT) allows real-time imaging performances that disclose great possibilities for medical application. In order to attain the image in the PA signal, the image reconstruction approach plays a major part. Traditional noniterative reconstruction approaches, such as filtered back-projection (FBP), are widespread because of their fast speed. But, the imperfection delay-and-sum (DAS) of traditional approaches exist several artifacts that lead to distorted images, particularly in restricted view configuration [3–5]. In such cases, the iterative methods are well-adopted when using an appropriate regularization. PAT is a noninvasive combined physics biomedical imaging approach that conveniently integrates the higher contrast of pure optical imaging through the higher spatial resolution of ultrasound imaging [6]. It can be dependent upon the generation of acoustic waves through illuminating a semitransparent biological or clinical object with a shorter optical pulse. The induced time-based acoustic wave is measured outside of the samples using an acoustic detector, and the measured information is utilized for recovering an image of the interior [7].

Vessel segmentation is a fundamental process for analyzing biomedical imaging. Hence, it is complicated for providing accurate and reliable vessel segmentation on PA imaging. [8] shows better segmentation performance on the blood vessels taken from photoacoustic computed tomography (PACT). Optical resolution photoacoustic microscopy (OR-PAM) features simple imaging reconstruction and higher spatial resolution [9] and therefore has initiated broad application in vascular imaging. Up till now, several attempts have focused on the segmentation of the blood vessel taken by the OR-PAM method. Image segmentation has been considered an essential task in image processing and plays a significant part in image research in various areas of application such as pattern recognition, computer vision, medical imaging, robotic vision, cryptography, and agriculture [10]. When the results of segmentation are not correct, the classification approach might fail. Multiple challenges have been widely resolved by the segmentation method. The major purpose of segmentation is to split an image into many homogeneous segments/regions with common features such as color, texture, contrast, brightness, size, and form depending upon certain thresholding value(s) [11]. Often, it is extensively employed for distinguishing background and foreground as the primary phase for interpreting and identifying images.

Various methodologies, like threshold-based, region-based, feature-based, and edge-based clustering, were proposed for solving present challenges and enhance quality of the research. The most popular image segmentation methods are multilevel thresholding that tries to group the pixel which allocates features to a finite amount of pixels. Image thresholding methods are easier to produce and implement efficient than segmentation performances [12]. Thresholding comprises 2 classes: bi and multilevel. A single threshold value is utilized in the preliminary class for separating the images into 2 homogeneous background and foreground regions. The latter methods are employed for segmenting an image into more than 2 areas according to the pixel intensity called a histogram. Therefore, it can be expressed as an optimization issue with parametric/nonparametric methods.

Since the vessel segmentation on PAI is a difficult process, this paper employs metaheuristic optimization-based vascular segmentation techniques for PAI. This study employs two different types of vessel segmentation techniques such as Shannon's entropy function and Otsu thresholding. In addition, the threshold value and entropy function in the segmentation process are optimized using three metaheuristics such as the cuckoo search (CS), equilibrium optimizer (EO), and harmony search (HS) algorithms. For examining the improved performance of the proposed model, a wide range of simulations take place and the results are inspected under various aspects.

The rest of the paper is organized as follows: Section 2 offers the related works, Section 3 provides the proposed model, Section 4 gives the experimental validation, and Section 5 concludes the study.

## 2. Literature Review

Luke et al. [13] proposed a novel deep neural network (DNN) method for concurrently estimating the oxygen saturation in blood vessels and segmenting the vessels in the nearby background tissues. The network was trained on evaluating early pressure distribution from a 3D Monte Carlo simulation of light transport in breast tissues. Bench et al. [14] illustrate the capacity of DNN for processing entire three-dimensional images and outputting three-dimensional maps of vascular sO2 from realistic tissue images or models. The 2 separate fully convolutional neural network (FCNN) methods have been trained for producing three-dimensional maps of vascular blood oxygen saturation and vessel position from multiwavelength simulated images of tissue models.

In [15], a novel segmentation system is developed for PAI which directly produces an assessment of its consistency. Segmentation based on parameters has a natural tendency to improve consistency as the parameter value monotonically changes. The consistency is measured by calculating the classification of an image voxel with distinct parameters. In [16], U-net and FCN have been individually employed in PA imaging for vascular segmentation, and a hybrid network consists of both that is integrated by a voting system on the PA vascular image. The result is qualitatively related and estimated on intersection of union (IoU), dice coefficients, accuracy, and sensitivity.

In [17], tumor vessel is quantified and segmented in a whole three-dimensional framework. It has been tested from the phantom experiment that the three-dimensional quantification outcomes have higher performance when compared to two dimensions. Furthermore, in vivo vessel images have been quantified with two- and three-dimensional quantification systems correspondingly. Additionally, the variation between these 2 outcomes is important. Zhao et al. [18] presented an approach adoptive to the microvascular segmentation in photoacoustic image that includes morphological connection and a Hessian matrix enhancement operator. The precision of these vascular segmentation approaches is quantitatively estimated by several criteria. In order to attain continuous and accurate microvascular skeletons, an enhanced skeleton extraction-based multistencil fast marching (MSFM) approach has been presented.

Boink et al. [19] suggest conjointly obtaining the photoacoustic segmentation and reconstruction by altering a newly presented partially learned model based on the CNN method. They examine the stability of the method against modifications in early pressure and photoacoustic system settings. This insight is utilized for developing a method, i.e., strong input and system setting. This method could simply be employed in other imaging models and adapted to implement other higher-level processes dissimilar to segmentation. Feng et al. [20] proposed a vivo experiment and numerical simulation on human subjects for investigating the opportunity of segmentation of photoacoustic signals and ultrasound guided detection from bone tissues in vivo in a noninvasive method.

## 3. Materials and Methods

**Figure 1:**
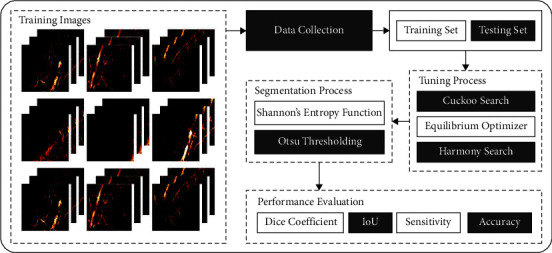
Overall process of the proposed method.

In this study, a set of metaheuristic optimization-based vascular segmentation techniques have been developed for PAI. The goal of the study is to inspect the outcome of the dissimilar metaheuristics on two segmentation approaches. In order to successfully segment the vessels, the SEF and MLOT techniques are applied. Furthermore, three optimization algorithms such as CS, EO, and HS are applied for optimal choice of entropy function and threshold values.  Figure 1 demonstrates the overall block diagram of the proposed method.

### 3.1. Image Segmentation Approach

In this study, SEF and MLOT techniques were applied to segment the vessels on PAI. The detailed workings of these two segmentation approaches are provided in the following:

#### 3.1.1. Design of Shannon's Entropy Function

Shannon's entropy model is an important concept from the field of “information theory and coding.” This theory has been utilized for probabilistically determining the count of data broadcast with some data. Consider that an image is a (*k*+1) homogeneous area with a *k* threshold gray level at *t*_1_, *t*_2_, *t*_3_,…, *t*_*k*__,_ then the following equation is obtained:

(1) $$\begin{array}{c} h\left(i\right)=\frac{f_{i}}{N}\;,\;i=0,1,2,\;,\;255, \end{array}$$

where *f*_*i*_ represents the frequency of *i*^*th*^ gray levels, *N* implies the entire amount of gray levels existing from the images, and *h*(*i*) refers the normalization frequency. The Shannon entropy function has been defined as follows [21]:

(2) $$\begin{array}{c} H=-\sum_{i=0}^{t_{1}}P_{1i}\ln\left(P_{1i}\right)-\sum_{i=t_{1}+1}^{t_{2}}P_{2i}\ln\left(P_{2i}\right)-\cdots -\sum_{i=t_{k}}^{255}P_{ki}\ln\left(P_{ki}\right), \end{array}$$

where

(3) $$\begin{array}{c} P_{1i}=\frac{h\left(i\right)}{\sum_{i=0}^{t_{1}}h\left(i\right)},\;\text{ for }0\leq i\leq t_{1},\\ P_{2i}=\frac{h\left(i\right)}{\sum_{i=t_{1}+1}^{t_{2}}h\left(i\right)}\;,\;\text{ for }t_{1}+1\leq i\leq t_{2},\\ P_{ki}=\frac{h\left(i\right)}{\sum_{i=t_{k}+1}^{255}h\left(i\right)},\;\text{ for }t_{k}+1\leq i\leq 255. \end{array}$$

The Shannon entropy function *H* from equation (1) has been executed as an objective function that can be maximized using the metaheuristic optimization algorithms such as CS, HS, and EO algorithms.

#### 3.1.2. Design of Multilevel Ostu Thresholding (MLOT) Technique

Otsu [22] is a widely employed segmentation approach utilized for finding a better threshold value of an image dependent upon maximizing the between-class variance. These techniques were utilized for finding the threshold optimal value which split the image into several classes. This technique recognizes the *L*_*v*_ intensity level of gray images and the probability distribution has been estimated in equation (2). It could be utilized to color images, in that Otsu has been implemented to all channels.

(4) $$\begin{array}{c} h_{i}=\frac{h_{i}}{NP},\\ \sum_{i=1}^{NP}Ph_{i}=1, \end{array}$$

where *i*_*l*_ refers the intensity level identified from the range of (0 ≤ *i*_*l*_ ≤ *L*_*v*_ − 1). *NP* signifies the entire amount of image pixels. *h*_*i*_indicates the amount of the presence of intensity *i*_*l*_ under the image signified as the histograms. The histogram has been normalized from the probability distributions of *Ph*_*i*_. According to the probability distribution/thresholding value (*th*), the classes have been defined to bilevel segmentation as follows:

(5) $$\begin{array}{c} C_{1}=\frac{Ph_{1}}{\omega_{0}\left(th\right)},\ldots ,\frac{Ph_{th}}{\omega_{0}\left(th\right)}\text{ and }C_{2}=\frac{Ph_{th+1}^{c}}{\omega_{1}\left(th\right)},\ldots ,\frac{Ph_{L}}{\omega_{1}\left(th\right)}, \end{array}$$

where *ω*_0_(*th*) and *ω*_1_(*th*) are cumulative probability distribution to *C*_1_ and *C*_2_, respectively, as it can be demonstrated in equation (4).

(6) $$\begin{array}{c} \omega_{0}\left(th\right)=\sum_{i=1}^{th}Ph_{i}\text{ and }\omega_{1}\left(th\right)=\sum_{th+1}^{L}Ph_{i}. \end{array}$$

It can be required for finding the average intensity levels *μ*_0_ and *μ*_1_ which are utilized in equation (5). If these values are *c*, the Otsu based on class *σ*_*B*_^2^ is determined in equation (6).

(7) $$\begin{array}{c} \mu_{0}=\sum_{i=1}^{th}\frac{iPh_{i}}{\omega_{0}\left(th\right)}\;\text{ and }\mu_{1}=\sum_{i=th+1}^{L}\frac{iPh_{i}}{\omega_{1}\left(th\right)},\\ \sigma_{B}^{2}=\sigma_{1}+\sigma_{2}. \end{array}$$

Noticeable *σ*_1_ and *σ*_2_ in equation (6) are the differences of *C*_1_ and *C*_2_ which are identified as follows:

(8) $$\begin{array}{c} \sigma_{1}=\omega_{0}{\left(\mu_{0}+\mu_{T}\right)}^{2}\text{ and }\sigma_{2}=\omega_{1}{\left(\mu_{1}+\mu_{T}\right)}^{2}, \end{array}$$

where *μ*_*T*_=*ω*_0_*μ*_0_+*ω*_1_*μ*_1_ and *ω*_0_+*ω*_1_=1 are dependent upon the values *σ*_1_ and *σ*_2_, and equation (8) offers the objective functions. So, the optimized issue has been decreased for finding the intensity level that maximizes in equation (8).

(9) $$\begin{array}{c} F_{otsu}\left(th\right)=\max\left(\sigma_{B}^{2}\left(th\right)\right)\text{ where }0\leq th\leq L-1, \end{array}$$

where *σ*_*B*_^2^(*th*) stands for Otsu's alteration to provided *th* value. An objective function *F*_*otsu*_(*th*) in equation (8) is also altered to several thresholds as follows:

(10) $$\begin{array}{c} F_{otsu}\left(TH\right)=\text{Max}\left(\right)\sigma_{B}^{2}\left(th\right)\;where\;0\leq th_{i}\leq L-1\;an\;d\;i=\left[1,2,3,\;\ldots ,\;k\right]\;\left(9\right), \end{array}$$

where *TH*=[*th*_1_,  *th*_2_,   …,  *th*_*k*_ − 1] has been a vector comprising several thresholds, *L* refers to the maximal gray level, but the alterations are computed in equation (10).

(11) $$\begin{array}{c} \sigma_{B}^{2}=\sum_{i=1}^{k}\sigma_{i}\\ =\sum_{i=1}^{k}\omega_{1}{\left(\mu_{1}-\mu_{T}\right)}^{2}, \end{array}$$

where *i* demonstrates the particular class, and *ω*_*i*_ and *μ*_*j*_ implies the probability of existence and the mean of level, respectively. Multilevel thresholding values are attained as follows:

(12) $$\begin{array}{c} \omega_{k-1}\left(th\right)=\sum_{i=th_{k}+1}^{L}Ph_{i}. \end{array}$$

With respect to the mean values as defined in equation (12), the followingequationis obtained:

(13) $$\begin{array}{c} \mu_{k-1}=\sum_{i=th_{k}+1}^{L}\frac{iPh_{i}}{\omega_{1}\left(th_{k}\right)}. \end{array}$$

In this study, the threshold values can be optimally chosen by the use of three optimization algorithms, namely, CS, HS, and EO algorithms.

### 3.2. Metaheuristic Optimization Models

In this section, the algorithmic procedures of the CS, EO, and HS algorithms are elaborated in detail. These algorithms are employed for the proficient selection of the entropy function in SEF and the threshold value in the MLOT technique.

#### 3.2.1. Process Involved in the CS Algorithm

The CS technique is simulated by the social performances of cuckoos. The CS is a population-based search technique for identifying an optimum result of optimized issues. A novel set of experimental results utilizing this technique tries to determine, on previous initiation, the optimum experimental results. The general framework and competence to explain these issues with distinct execution can be carried out with the CS technique. The CS has been established in concern of the social pensive ability of the cuckoo bird. Generally, the cuckoo bird places its eggs from the nest of another alien bird. The alien bird is declaring that the egg has the associated probability of *pa* ∈ [0,1]. Therefore, it is also the egg that has been thrown in the nest or forsaken which nest to construct a novel one with the alien bird. Thus, all the eggs of the alien nest carried out a solution.

Mathematically, the CS algorithm is expressed in three major levels. Initially, the accessibility of the host nest was set. The next posit is that all cuckoos locate only a single egg at a timestamp for a randomly chosen host nest, and finally, the maximum quality eggs from the optimum nest are surplus to the next generations [23]. Assume that *X*_*i*_(*t*) implies the current search space of cuckoo *iwherei* = 1,  2,   ⋯ ,  *N* at a time *t*, represented as *X*_*i*_=(*x*_*i*_^1^, *x*_*i*_^2^,   … …,  *x*_*i*_^*n*^) from the *n* dimension quandary. Afterward, the initial search space *X*_*i*_(*t*+1) to the later generations at time *t*+1 has been mathematically computed as follows:

(14) $$\begin{array}{c} X_{i}\left(t+1\right)=X_{i}\left(t\right)+\alpha Levy\left(\lambda \right), \end{array}$$

where *α* > 0 represents the step-size that is similar to the scales of the quandary of the curiosity. In maximal cases *α* is generally taken as 1.

It offers a random walk, and their random steps have been drawn in Levy distribution to immensely colossal steps that are demonstrated as follows:

(15) $$\begin{array}{c} Levy\;\left(\lambda \right)u=t^{-\lambda },\;\text{where }1<\lambda <3. \end{array}$$

The 2 very famous techniques are Mantegna's technique and McCulloch's technique. The Levy step size has been attained in the Mantegna technique as initialized, which is as follows:

(16) $$\begin{array}{c} Levy=\frac{v}{{\left|\nu \right|}^{1/\left(\lambda -1\right)}}. \end{array}$$

**Figure 2:**
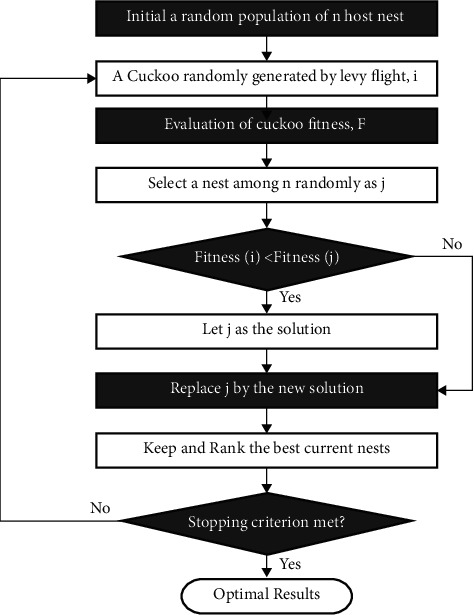
Flowchart of the CS algorithm.

The conditions for forsake probabilities, the entire size of populations, and the maximal number of cuckoos reproduced in a lifetime are fixed to the utilizer; however, primary terms at the starting has been reached.  Figure 2 depicts the flowchart of the CS technique.

Assume that *t* symbolizes the current generation, *t*_max_ signifies the determined greatest span of animating being life redundancy, and that the cuckoo influence roams from the lifetime. Therefore, an *n*-dimension issue to cuckoo at the starting generation *t*=1 is that set up and it can be represented in equation (16).

(17) $$\begin{array}{c} x_{1}^{n}\left(t=1\right)=r\text{and}n\times \left({\text{Upper}}^{n}-{\text{Lower}}^{n}\right), \end{array}$$

where Lower^*n*^ and Upper^*n*^ refer the lowermost as well as uppermost outer restricts of the search space of *n* th attributes, respectively. These maintain the mastery capable time-optimized technique to stay within the individual perimeter.

In equation (15), *υ* and *ν* demonstrate the taken in normal distributions. That means *υ*  ~ *N*(0,  *δ*^2^) and *ν*  ~ *N*(0,1) with the following:

(18) $$\begin{array}{c} \delta ={\left(\frac{\Gamma \left(1+\beta \right)\sin\left(\pi \pi /2\right)}{\Gamma \left(1+\beta /2\right)\beta \times 2^{\left(\left(\beta -1\right)/2\right)}}\right)}^{1/\beta }, \end{array}$$

where Γ stands for the gamma function, and it can be demonstrated as follows:

(19) $$\begin{array}{c} \Gamma \left(p\right)=\int_{0}e^{-t}t^{p-1}\text{d}t\&\beta \in \left[0,2\right]\text{ is a scale factor}. \end{array}$$

#### 3.2.2. Process Involved in the EO Algorithm

EO follows a dynamic mass balance process that works to control volume. The mathematical formula has been utilized for representing the mass balance for defining the focus of nonreactive components in the dynamic environments of control volume and this formula as a function with their different processes in the changes of source and sink. The entire theoretical explanation of EO according to these terms is defined as follows:

The arbitrary population (primary concentration) was initialized by utilizing a uniform distribution dependent upon the amount of particles and dimension in the provided search region as follows:

(20) $$\begin{array}{c} C_{i}^{\text{initial}}=C_{\min}+rand_{i}\left(C_{\max}-C_{\min}\right)i=1,2,\ldots ,\;n, \end{array}$$

where *C*_*i*_^initial^ represents the vector of primary concentration of *ith* particle, *C*_min_ and *C*_max_ refer the lower as well as upper bounds, *rand*_*i*_ signifies the uniform arbitrary numbers created in intervals from 0 to 1, and *n* defines the size of populations.

In order to determine the equilibrium state (global optimum), a pool of 4 optimum candidates is identified, that comprises of particles with a concentration identical to arithmetic mean of 4 particles. These particles produced a pool vector, as ffgiven in (20).

(21) $$\begin{array}{c} {\overset{⟶}{C}}_{eq.\text{pool}}=\left\{{\overset{⟶}{C}}_{eq\left(1\right)},{\overset{⟶}{C}}_{eq\left(2\right)},{\overset{⟶}{C}}_{eq\left(3\right)},{\overset{⟶}{C}}_{eq\left(4\right)},{\overset{⟶}{C}}_{eq\left(ave\right)}\right\}. \end{array}$$

In the evolution procedure, initial particles upgrade their concentration from the initial generation dependent upon ${\overset{⟶}{C}}_{eq\left(1\right)}$, and then in the second generation, the upgrading can take place on ${\overset{⟶}{C}}_{eq\left(ave\right)}$. Afterward, all the particles with every candidate solution have been upgraded by the completion of the evolution procedure.

The exponential term *F* demonstrates that it assists EO with attaining the appropriate balance amongst diversification as well as intensification [24]. The term *λ* has been an arbitrary value from the intervals 0 and 1 for controlling the turnover rate from real control volume.

(22) $$\begin{array}{c} \vec{F}=e^{-\vec{\lambda }\left(t-t_{0}\right)}, \end{array}$$

where *t* has been provided as function of the amount of iteration (*Iter*) and is determined as follows:

(23) $$\begin{array}{c} t=\left(1-\frac{Iter}{{\text{Max}}_{-}iter}\right)\left(a_{2}\frac{Iter}{{\text{Max}}_{-}iter}\right), \end{array}$$

where *Iter*=current iteration, Max_*iter*= maximal iteration, and parameter *a*_2_ was employed for controlling exploitation capability of *EO*. For ensuring convergence while improving global as well as local search capability of the technique, in written form is also employed as follows:

(24) $$\begin{array}{c} \vec{t_{0}}=\frac{1}{\vec{\lambda }}\;\ln\left(-a_{1}sign\left(\vec{r}-0.5\right)\left[1-e^{-\vec{\lambda }t}\right]\right)+t, \end{array}$$

where *a*_1_ and *a*_2_  have been utilized for controlling global as well as local search capability of the EO technique. The terms *sign*$\left(\vec{r}-0.5\right)$ have been responsible of the nearby path of explorations as well as exploitations. In *EO*, the values of *a*_1_ and *a*_2_ are elected to be 2 and 1, respectively.

With replacing equation (23) in (21), the terms are altered as follows:

(25) $$\begin{array}{c} \vec{F}=a_{1}\text{ sign }\left(\vec{r}-0.5\right)\left[e^{-\vec{\lambda }t}-1\right]. \end{array}$$

The generation rate in the EO technique was implemented for improving exploitation that is employed as a function of time. The 1^st^ order exponential decay procedure from the method of generation rate of a multipurpose technique is determined as follows:

(26) $$\begin{array}{c} \vec{G}={\vec{G}}_{0}e^{-\vec{k}\left(t-t_{0}\right)}, \end{array}$$

where *G*_0_= primary value and *k*= decay parameters.

Eventually, the generation rate appearance considering *k*=*λ* has been determined as follows:

(27) $$\begin{array}{c} \vec{G}={\vec{G}}_{0}e^{-\vec{\lambda }\left(t-t_{0}\right)},\\ ={\vec{G}}_{0}{\vec{F}}_{0}. \end{array}$$

In equation (26), *G*_0_ has been estimated as follows:

(28) $$\begin{array}{c} {\vec{G}}_{0}=G\vec{C}P\left({\vec{C}}_{eq}-\vec{\lambda }\vec{C}\right),\\ G\vec{C}P=\left\{\begin{array}{cc} 0.5r_{1}, & r_{2}\geq 0,\\ 0, & r_{2}<0, \end{array}\right) \end{array}$$

where *r*_1_, *r*_2_ demonstrate 2 arbitrary numbers from intervals 0 and 1 and *GCP* denotes generation rate control parameter control generation rate. By means of all the abovementioned expressions, the last upgrade formula of concentration (particle) is determined as follows:

(29) $$\begin{array}{c} \vec{C}={\vec{C}}_{eq}+\left(\vec{C}-{\vec{C}}_{eq}\right)\vec{F}\\ +\frac{\vec{G}}{\vec{\lambda }V}\left(1-\vec{F}\right). \end{array}$$

The upgraded formula has 3 terms: 1st term is equilibrium concentration; the 2nd term is employed to global search; and 3rd term is in charge of local search for achieving exact solution.

#### 3.2.3. Process Involved in the HS Algorithm

In HSA, the feasible solution (or element from the solution spaces) is called “harmony” that is an n-dimension real vector whose arbitrary values have been allocated to the primary population and loaded from harmony memory (HM). During the next step, an estimated novel candidate (nest generation/iteration) harmony was formed regarded as the element from HM, either by changing the pitch or by arbitrarily choosing to upgrade the element from HM. As the last step, the element from the harmony memory is correlated with the least HM vector and recently calculated candidate harmony, and the entire procedure is repeated an amount of times for satisfying the end condition.

The HS optimization technique parameters are as follows: (i) size of HM, (ii) HM consideration rate (HMCR), (iii) pitch adjusting rate (PAR), and (iv) distance bandwidth (BW), and the number of iterations or improvisations (NI). During this step, it can be vital for configuring the primary HM modules (HMS vectors). Assume that *xi*={*xi*(1), *xi*(2),…*xi*(*n*)} represents an arbitrarily calculated HM vector: *xi*(*k*)=*X*_*l*_(*k*)+(*X*_*u*_(*k*) − *X*_*l*_(*k*))*∗* rand (0,1) for  *k*=1,2,…,  nandi=1,2,…, *HMS* which is the length of HM. So, the lower as well as upper restricts of feasible search space are represented as *X*_*l*_(*k*) and *X*_*u*_(*k*), respectively [25]. Next, in the HM matrix, all components are a harmony vector, so it can be that HMS has a vector present.

(30) $$\begin{array}{c} HM=\left[\begin{array}{c} x_{1}\\ x_{2}\\ \vdots \\ x_{HMS} \end{array}\right]. \end{array}$$

In the HM matrix, *x*_new_, a novel harmony vector, has been created utilizing 3 functions: (i) memory consideration, (ii) arbitrary reinitialization, and (iii) pitch adjustment. During the initial stage, the primary decision value *x*_new_(1) was chosen arbitrarily in harmony provided as {*x*_1_(1), *x*_2_(1) … .*x*_*HMS*_(1)}. In order to choose *x*_new_(1) an arbitrary number *r*1 with a range of 0 and 1, when this arbitrary number has been under HMCR, *x*_new_(1) has been created as a memory consideration, otherwise *x*_new_(1) has been captured in the search range [*X*_*l*_(*k*), *X*_*u*_(*k*)]. In the same manner, *x*_new_(2), *x*_new_(3),……, *x*_new_(*n*) are chosen so that the 2 functions such as memory consideration and arbitrary reinitialization are determined as follows:

(31) $$\begin{array}{c} x_{new}\left(k\right)=\left\{\begin{array}{c} x_{i}\in \left\{x_{1}\left(k\right)\ldots \ldots \ldots \ldots .x_{HMS}\left(k\right)\right\},\\ X_{l}\left(k\right)+\left(X_{u}\left(k\right)-X_{l}\left(k\right)\right)*ran\;d\left(0,1\right),\\ \text{with probability of }1‐HMCR. \end{array}\right) \end{array}$$

All recently created *x*_new_ values were examined to determine whether they can be pitch adjusted or not. To resolve this, the pitch adjustment rate (PAR) has been developed which is a composition of frequency and bandwidth factor (BF). These 2 factors are modified to obtain a novel *x*_new_ value in local search for the chosen solutions of HM. The pitch modified novel solution *x*_new_ has been computed as *x*_new_(*k*)+/− rand(0,1) · *BW* with probabilities PAR. This pitch adjustment is most same as the mutation procedure used in some evolutionary bioinspired techniques. The range of pitch adjustment has been restricted as [*X*_*l*_(*k*), *X*_*u*_(*k*)]_._

From the last phase, *x*_new_, a novel harmony vector created, the HM has been estimated/upgraded as a novel completion to fitness amongst *x*_new_ and a worse harmony vector *x*_*w*_ in the HM. Accordingly *x*_*w*_ has been changed by *x*_new_ and developed as part of HM. To maximize the objective function (interclass variance), HSA has been utilized.

The harmony or solution utilizes *k* distinct elements for deciding the optimized technique. These variables have been threshold values *th*_*k*_ which is more utilized to multilevel segmentation. The population of the technique has been expressed as follows:

(32) $$\begin{array}{c} HM={\left[x_{1},x_{2}\ldots ,x_{1HMS}\right]}^{T},\\ x_{i}=\left[th_{1},\;th_{2}\ldots \ldots th_{k}\right]. \end{array}$$

**Figure 3:**
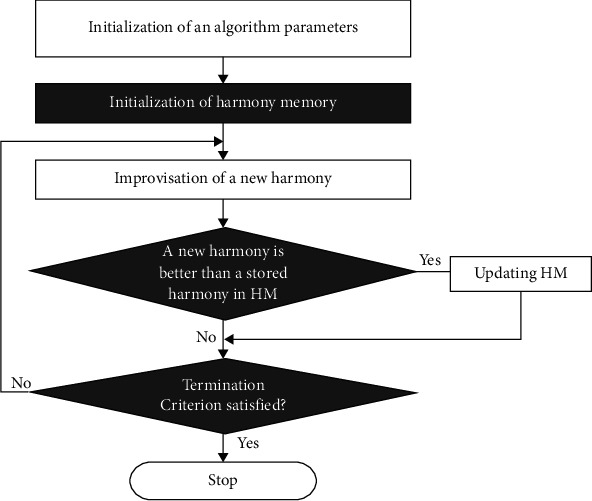
Flowchart of the HS algorithm.

In the abovementioned formula *T* represents the transpose of the matrix, the maximal size of HM has been represented as HMS, and all the elements from HM have been demonstrated as *xi*, where the range of *i* is [0, *k*].  Figure 3 demonstrates the flowchart of the HS technique.

## 4. Experimental Validation

**Figure 4:**
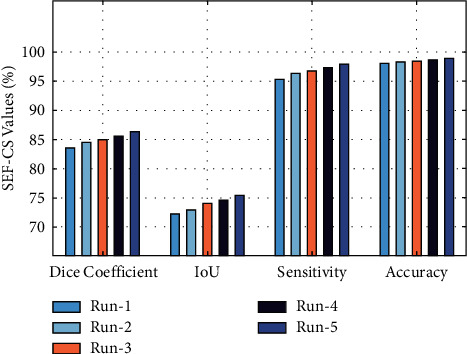
Result analysis of the SEF-CS model with different runs.

Table 1 provides the results analysis of the SEF with three optimization algorithms under five distinct runs.  Figure 4 showcases the results analysis of the SEF-CS technique on five test runs. The results demonstrated that the SEF-CS technique has accomplished maximum performance under all runs. For instance, with run-1, the SEF-CS technique has attained a dice coefficient of 84.89%, an IoU of 73.92%, a sensitivity of 96.76%, and an accuracy of 98.38%, respectively. In addition, under run-3, the SEF-CS technique has offered a dice coefficient of 83.52%, an IoU of 72.32%, a sensitivity of 95.26%, and an accuracy of 98.07%, respectively. Moreover, the SEF-CS technique has provided an average dice coefficient of 84.92%, an IoU of 73.82%, a sensitivity of 96.66%, and an accuracy of 98.46%, respectively.

**Figure 5:**
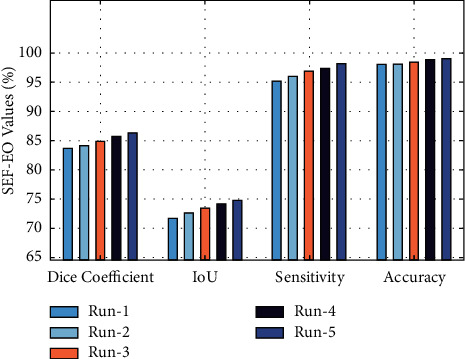
Result analysis of the SEF-EO model with different runs.

Figure 5 illustrates the results analysis of the SEF-EO technique on five test runs. The results demonstrated that the SEF-EO technique has accomplished maximum performance under all runs. For instance, with run-1, the SEF-EO technique has attained a dice coefficient of 83.77%, an IoU of 71.75%, a sensitivity of 95.26%, and an accuracy of 98.09%, respectively. Besides, under run-3, the SEF-EO approach has offered a dice coefficient of 84.93%, an IoU of 73.55%, a sensitivity of 96.87%, and an accuracy of 98.49%, respectively. Furthermore, the SEF-EO technique has provided an average dice coefficient of 85.44%, an IoU of 73.39%, a sensitivity of 96.76%, and an accuracy of 98.55%, correspondingly.

**Figure 6:**
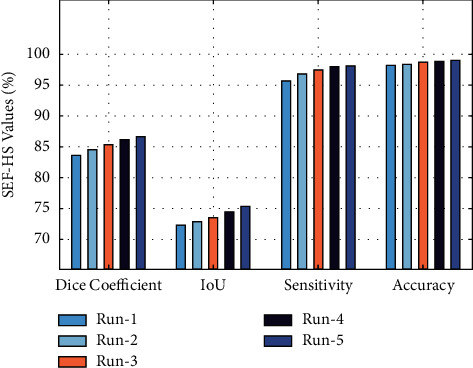
Result analysis of the SEF-HS model with different runs.

Figure 6 depicts the results analysis of the SEF-HS technique on five test runs. The results demonstrated that the SEF-HS technique has accomplished maximum performance under all runs. For instance, with run-1, the SEF-HS technique has attained a dice coefficient of 83.59%, an IoU of 72.25%, a sensitivity of 95.65%, and an accuracy of 98.18%, respectively. At the same time, under run-3, the SEF-HS technique has offered a dice coefficient of 85.45%, an IoU of 73.55%, a sensitivity of 97.55%, and an accuracy of 98.69%, respectively. Finally, the SEF-HS technique has provided an average dice coefficient of 85.27%, an IoU of 73.69%, a sensitivity of 97.23%, and an accuracy of 98.62%, correspondingly.

**Figure 7:**
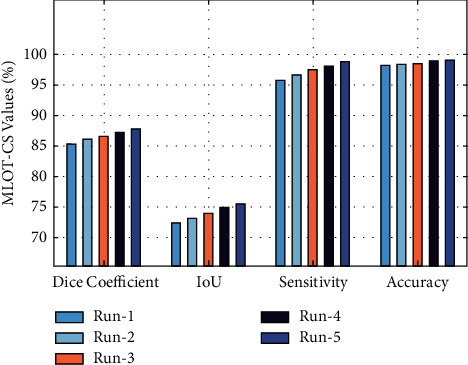
Result analysis of the MLOT-CS model with different runs.

Table 2 provides the results analysis of the MLOT with three optimization techniques under five distinct runs.  Figure 7 demonstrates the results analysis of the MLOT-CS technique on five test runs. The results demonstrated that the MLOT-CS technique has accomplished maximum performance under all runs. For instance, with run-1, the MLOT-CS technique has attained a dice coefficient of 85.31%, an IoU of 72.25%, a sensitivity of 95.80%, and an accuracy of 98.25%, respectively. Likewise, under run-3, the MLOT-CS technique has offered a dice coefficient of 86.51%, an IoU of 73.82%, a sensitivity of 97.60%, and an accuracy of 98.55%, respectively. Eventually, the MLOT-CS technique has provided an average dice coefficient of 86.55%, an IoU of 73.83%, a sensitivity of 97.44%, and an accuracy of 98.67%, respectively.

**Figure 8:**
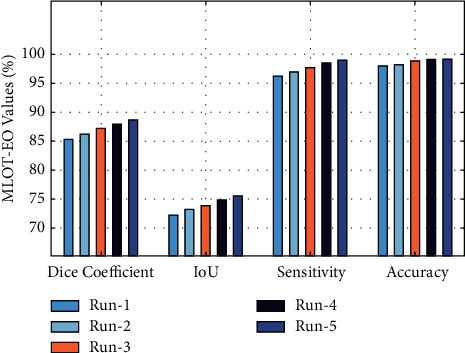
Result analysis of the MLOT-EO model with different runs.

Figure 8 showcases the results analysis of the MLOT-EO technique on five test runs. The results demonstrated that the MLOT-EO technique has accomplished maximum performance under all runs. For instance, with run-1, the MLOT-EO technique has attained a dice coefficient of 85.31%, an IoU of 72.25%, a sensitivity of 96.15%, and an accuracy of 98%, respectively. Moreover, under run-3, the MLOT-EO technique has offered a dice coefficient of 87.21%, an IoU of 78.85%, a sensitivity of 97.65%, and an accuracy of 98.18%, respectively. Then, the MLOT-EO technique provided an average dice coefficient of 87.07%, an IoU of 73.97%, a sensitivity of 97.63%, and an accuracy of 98.64%, correspondingly.

**Figure 9:**
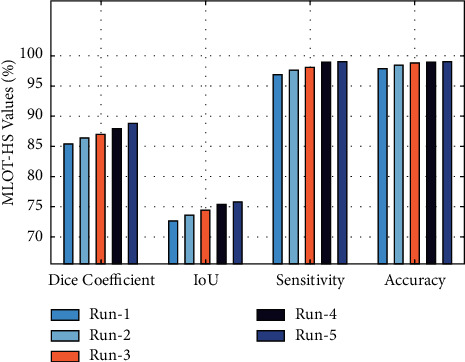
Result analysis of the MLOT-HS model with different runs.

Figure 9 exhibits the results analysis of the MLOT-HS technique on five test runs. The results demonstrated that the MLOT-HS technique has accomplished maximum performance under all runs. For instance, with run-1, the MLOT-HS technique has attained a dice coefficient of 85.41%, an IoU of 72.60%, a sensitivity of 96.97%, and an accuracy of 98.01%, respectively. Followed by, under run-3, the MLOT-HS technique has offered a dice coefficient of 86.91%, an IoU of 74.30%, a sensitivity of 98.12%, and an accuracy of 98.90%, respectively. Finally, the MLOT-HS technique has provided an average dice coefficient of 87.09%, an IoU of 74.30%, a sensitivity of 98.22%, and an accuracy of 98.71%, correspondingly.

**Figure 10:**
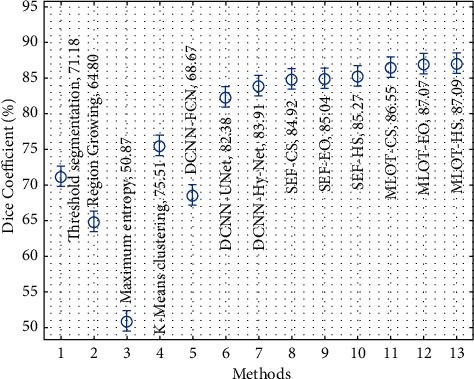
Comparative analysis of proposed method interms of dice coefficient.

Finally, a comprehensive comparison study of the proposed and existing techniques takes place in Table 3.  Figure 10 investigates the dice coefficient analysis of the proposed with existing techniques. The results show that the maximum entropy, region growing, DCNN-FCN, thresholding segmentation, and K-means clustering techniques have accomplished ineffective outcomes compared to the other techniques. At the same time, the DCNN-UNet, DCNN-Hy-Net, SEF-CS, SEF-EO, and SEF-HS techniques have accomplished moderately closer dice coefficient values. Followed by the MLOT-CS and MLOT-EO techniques, they have resulted in certainly improved dice coefficient values of 86.55% and 87.07%, respectively. However, the MLOT-HS technique has gained effective performance with a higher dice coefficient of 87.09%.

**Figure 11:**
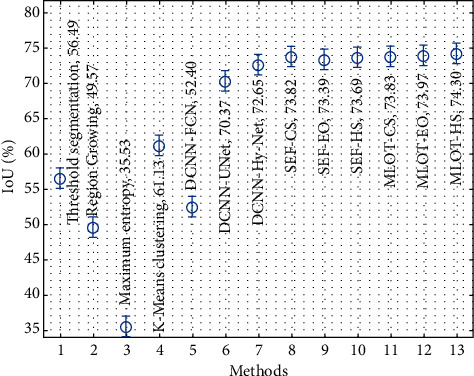
Comparative analysis of the proposed method in terms of IoU.

Figure 11 examines the IoU analysis of the proposed with existing techniques. The results show that the maximum entropy, region growing, DCNN-FCN, thresholding segmentation, and K-means clustering techniques have accomplished ineffective outcomes compared to the other techniques. In line with the DCNN-UNet, DCNN-Hy-Net, SEF-CS, SEF-EO, and SEF-HS techniques, they have accomplished moderately closer IoU values. Following that the MLOT-CS and MLOT-EO techniques have resulted to certainly improved IoU values of 73.83% and 73.97%, correspondingly. However, the MLOT-HS technique has gained effective performance with a higher IoU of 74.30%.

**Figure 12:**
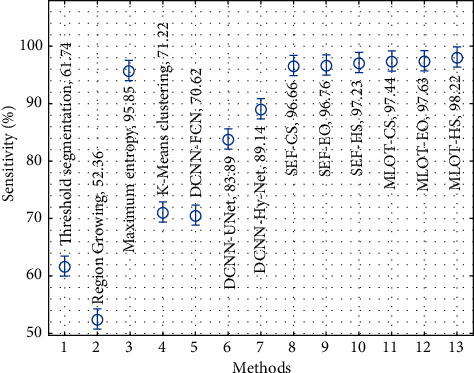
Comparative analysis of the proposed method in terms of sensitivity.

Figure 12 considers the sensitivity analysis of the proposed with existing techniques. The results show that the maximum entropy, region growing, DCNN-FCN, thresholding segmentation, and *K*-means clustering techniques have accomplished ineffective outcomes compared to the other techniques. Simultaneously, the DCNN-UNet, DCNN-Hy-Net, SEF-CS, SEF-EO, and SEF-HS techniques have accomplished moderately closer sensitivity values. Also, the MLOT-CS and MLOT-EO techniques have resulted to certainly improved sensitivity values of 97.44% and 97.63%, respectively. However, the MLOT-HS technique has gained effective performance with a higher sensitivity of 98.22%.

**Figure 13:**
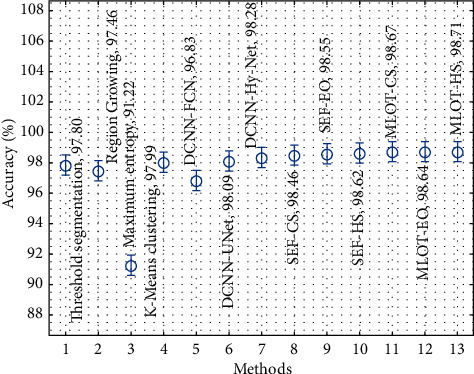
Comparative analysis of the proposed method in terms of accuracy.

Figure 13 explores the accuracy analysis of the proposed with existing techniques. The results show that the maximum entropy, region growing, DCNN-FCN, thresholding segmentation, and *K*-means clustering techniques have accomplished ineffective outcomes compared to the other techniques. Concurrently, the DCNN-UNet, DCNN-Hy-Net, SEF-CS, SEF-EO, and SEF-HS techniques have accomplished moderately closer accuracy values. Similarly, the MLOT-CS and MLOT-EO techniques have resulted to certainly improved accuracy values of 98.67% and 98.64%, respectively. Lastly, the MLOT-HS technique has gained effective performance with a higher accuracy of 98.71%. By looking into the abovementioned tables and figures, it is ensured that the MLOT-HS technique is found to be effective compared to other techniques.

## 5. Conclusion

In this study, a set of metaheuristic optimization-based vascular segmentation techniques have been developed for PAI. The goal of the study is to examine the outcomes of the different metaheuristics on two segmentation approaches. In order to effectively segment the vessels, the SEF and MLOT techniques are applied. Moreover, three optimization algorithms such as CS, EO, and HS are utilized for optimal selection of entropy function and threshold values. For examining the improved performance of the proposed model, a wide range of simulations take place and the results are inspected under various aspects. The experimental results pointed out that the proposed MLOT-HS technique has gained maximum performance over the other existing approaches. Thus, it can be employed in real-time brain imaging related applications such as brain and cancer imaging such as prostate cancer, breast cancer, and ovarian cancer. In the future, the vessel segmentation performance of the proposed model can be improved by the use of deep learning models.

## Figures and Tables

**Table 1:** Result analysis of SEF with three optimization algorithms

No. of runs	Dice coefficient	IoU	Sensitivity	Accuracy
*SEF-CS*				
Run-1	83.52	72.32	95.26	98.07
Run-2	84.37	72.92	96.26	98.28
Run-3	84.89	73.92	96.76	98.38
Run-4	85.54	74.52	97.26	98.68
Run-5	86.29	75.42	97.76	98.89
Average	84.92	73.82	96.66	98.46

*SEF-EO*				
Run-1	83.77	71.75	95.26	98.09
Run-2	84.34	72.65	96.04	98.15
Run-3	84.93	73.55	96.87	98.49
Run-4	85.72	74.15	97.41	98.88
Run-5	86.44	74.85	98.20	99.15
Average	85.04	73.39	96.76	98.55

*SEF-HS*				
Run-1	83.59	72.25	95.65	98.18
Run-2	84.52	72.85	96.85	98.35
Run-3	85.45	73.55	97.55	98.69
Run-4	86.17	74.45	97.95	98.88
Run-5	86.64	75.35	98.15	99.02
Average	85.27	73.69	97.23	98.62

**Table 2:** Result analysis of MLOT with three optimization algorithms.

No. of runs	Dice coefficient	IoU	Sensitivity	Accuracy
*MLOT-CS*				
Run-1	85.31	72.25	95.80	98.25
Run-2	86.01	72.93	96.70	98.45
Run-3	86.51	73.82	97.60	98.55
Run-4	87.11	74.81	98.20	98.95
Run-5	87.81	75.35	98.90	99.16
Average	86.55	73.83	97.44	98.67

*MLOT-EO*				
Run-1	85.31	72.25	96.15	98.00
Run-2	86.21	73.25	96.95	98.18
Run-3	87.21	73.85	97.65	98.78
Run-4	87.91	74.85	98.45	99.10
Run-5	88.71	75.65	98.96	99.14
Average	87.07	73.97	97.63	98.64

*MLOT-HS*				
Run-1	85.41	72.60	96.97	98.01
Run-2	86.41	73.50	97.73	98.54
Run-3	86.91	74.30	98.12	98.90
Run-4	87.91	75.30	99.05	98.97
Run-5	88.81	75.80	99.25	99.13
Average	87.09	74.30	98.22	98.71

**Table 3:** Comparative results analysis of proposed versus existing techniques.

Methods	Dice coefficient	IoU	Sensitivity	Accuracy
Threshold segmentation	71.18	56.49	61.74	97.80
Region growing	64.80	49.57	52.36	97.46
Maximum entropy	50.87	35.53	95.85	91.22
*K*-means clustering	75.51	61.13	71.22	97.99
DCNN-FCN	68.67	52.40	70.62	96.83
DCNN-UNet	82.38	70.37	83.89	98.09
DCNN-Hy-net	83.91	72.65	89.14	98.28
SEF-CS	84.92	73.82	96.66	98.46
SEF-EO	85.04	73.39	96.76	98.55
SEF-HS	85.27	73.69	97.23	98.62
MLOT-CS	86.55	73.83	97.44	98.67
MLOT-EO	87.07	73.97	97.63	98.64
MLOT-HS	87.09	74.30	98.22	98.71

## Data Availability

No datasets were generated during this study.
